# Diagnostic Performance of Positron Emission Tomography in the Assessment of Systemic Amyloidosis: A Systematic Review and Meta-Analysis

**DOI:** 10.31083/RCM37731

**Published:** 2025-09-23

**Authors:** Tao Zhu, Lu Xu, Hua Pang

**Affiliations:** ^1^Nuclear Medicine Department, The First Affiliated Hospital of Chongqing Medical University, 400016 Chongqing, China

**Keywords:** systemic amyloidosis, positron emission tomography, cardiac amyloidosis

## Abstract

**Background:**

Positron emission tomography (PET) imaging with radiotracers can detect amyloid deposits in multiple organs. We conducted a systematic review and meta-analysis to evaluate the diagnostic performance of PET in patients with systemic amyloidosis.

**Methods:**

We searched PubMed, Cochrane, Embase, and Web of Science databases using the following keywords: “systemic amyloidosis” and “PET”. Studies evaluating organ involvement in systemic amyloidosis using PET were included. The pooled relative risk (RR) values for each affected organ were calculated. Sensitivity, specificity, positive and negative likelihood ratios (LRs+ and LRs-), and diagnostic odds ratios (DORs) were individually calculated to assess cardiac involvement by PET, and a summary receiver operating characteristic (SROC) curve was generated. The diagnostic performance of PET was compared in separate subgroup analyses based on the type of radiotracer and amyloidosis subtype.

**Results:**

Among 10 studies, the pooled RR values for PET detecting organ involvement in the bone marrow, central nervous system (CNS), heart, lungs, muscles, pancreas, salivary glands, spleen, thyroid, and tongue were statistically significant. In the seven studies on cardiac involvement, the pooled sensitivity and specificity were 0.98 and 0.61, respectively, with an area under the curve (AUC) of 0.8954. Subgroup analysis showed ^124^I-Evuzamitide had the highest sensitivity (0.98), while ^11^C-Pittsburgh Compound-B (^11^C-PIB) had the highest specificity (0.84). PET imaging detected cardiac involvement in light chain amyloidosis (AL) more effectively than in transthyretin amyloidosis (ATTR), with a pooled RR of 0.79 (*p* = 0.004).

**Conclusion:**

PET imaging has significant clinical value in assessing organ involvement in systemic amyloidosis, particularly for the early detection of cardiac involvement.

## 1. Introduction

Systemic amyloidosis is a group of diseases characterized by the deposition of amyloid fibrils in the extracellular space due to the misfolding of precursor proteins. These deposits impair organ function either through mechanical disruption or direct toxic effects on cells [1]. The accumulation of amyloid proteins can lead to multi-organ dysfunction, with the heart and kidneys being the most commonly affected organs across all types of amyloidosis. In particular, the expansion of the extracellular space in the heart ultimately results in restrictive cardiomyopathy. Cardiac involvement is a key determinant of prognosis, often leading to adverse outcomes [2, 3]. Early diagnosis is crucial as delayed treatment can result in severe adverse consequences. To date, 42 amyloid fibril proteins have been identified, of which 14 appear exclusively as systemic deposits, 24 are seen only in local amyloid deposits, and 4 can appear as both types. The two most common forms of systemic amyloidosis are transthyretin amyloidosis (ATTR) and light chain amyloidosis (AL) [3, 4]. The presence of amyloid proteins is typically confirmed by biopsy of affected organs or tissue samples containing amyloid deposits. Currently, subcutaneous fat tissue biopsy is widely used in clinical practice due to its minimal invasiveness and ease of operation, and these tissue samples can also be used for biochemical typing of amyloid proteins [5]. However, biopsies have notable limitations, including relatively high invasiveness, high technical demand on operators, and the potential for serious complications. Additionally, biopsies can only assess amyloid deposits in limited areas of local organs [6].

The focus has always been on cardiac imaging examinations, as death in patients with heart involvement is the primary cause. Non-invasive imaging plays a key role in the diagnosis of cardiac amyloidosis (CA). Echocardiography and cardiac magnetic resonance imaging (MRI) can detect structural changes and functional impairments in the heart caused by amyloidosis [7]. Additionally, serological markers such as cardiac troponin and N-terminal pro-B-type natriuretic peptide (NT-proBNP), have certain significance in the diagnosis of amyloidosis [8]. At present, the most widely recognized molecular imaging technique for diagnosing ATTR-CA is scintigraphy using ^99m^Tc-labeled bone-seeking tracers that are derivatives of bisphosphonates, namely ^99m^Tc pyrophosphate (PYP), ^99m^Tc 3,3-diphosphono-1,2-propanodicarboxylic acid (DPD) [9], and ^99m^Tc hydroxymethylene diphosphonate (HMDP) [10]. However, these methods have corresponding limitations and can only evaluate amyloid deposits in a single organ. Nonetheless, the disease burden of amyloidosis is systemic. Therefore, whole-body amyloid imaging has emerged as a new, visual, noninvasive approach to assess systemic amyloidosis. ^123^I-labeled serum amyloid P component (SAP) scintigraphy can detect amyloid deposits in visceral organs but is unreliable for detecting cardiac involvement and is available in only a few centers globally [11]. Based on alterations in regional calcium homeostasis, sodium fluoride (NaF)-positron emission tomography (PET)/computed tomography (CT) may serve as a feasible non-invasive approach for differentiating between ATTR and AL amyloidosis [12].

With advancements in other radiotracers, tracers for diagnosing Alzheimer’s disease such as ^11^C-Pittsburgh Compound-B (^11^C-PIB) [6, 13, 14], ^18^F-Florbetapir [15, 16, 17, 18, 19], and ^18^F-Florbetaben [20] have been applied to detect systemic amyloid deposits, making whole-body imaging of amyloidosis increasingly feasible. These tracers can detect and assess amyloid deposits in most organs, especially the heart. ^124^I-Evuzamitide (^124^I-labeled peptide p5+14) [21] also offers an opportunity for noninvasive detection of systemic amyloidosis through PET imaging.

Based on the existing literature, this meta-analysis aims to conduct a comprehensive review of current research on the use of PET imaging techniques in detecting organ involvement in systemic amyloidosis, with a particular focus on the diagnostic performance of cardiac involvement. This will provide further evidence to support future clinical applications and help optimize early diagnosis and management of amyloidosis patients.

## 2. Methods

### 2.1 Search Strategy and Study Selection

The researchers conducted a comprehensive search of electronic databases, including PubMed, Cochrane, Embase, and Web of Science, from their earliest indexed date to May 19, 2024. The database search utilized keywords or phrases related to “systemic amyloidosis” and “PET”. Included studies were clinical studies reporting on the use of PET imaging to evaluate organ involvement in systemic amyloidosis. Review articles, case reports, editorials, conference abstracts, letters, animal studies, and *in vitro* research were excluded. If studies were conducted by the same research team, only the study with the most complete information or the largest sample size was included. Two researchers independently conducted the literature search, screening, and inclusion of eligible studies. Any discrepancies were resolved through discussion until a final decision was reached.

### 2.2 Data Extraction and Quality Assessments

The following information was collected and recorded for the included studies: first author, year of publication, number of patients, involved organs, amyloidosis type, amyloid imaging tracer used, and type of detection method. For studies that assessed cardiac involvement, the absolute numbers of true positives (TP), false positives (FP), true negatives (TN), and false negatives (FN) related to cardiac involvement were recorded separately. Each article was assessed for quality using the Quality Assessment of Diagnostic Accuracy Studies (QUADAS-2) tool. This quality assessment system evaluates included studies for risk of bias and applicability concerns. Four key domains are used to assess bias risk: patient selection, index test, reference standard, and flow and timing. Applicability concerns include patient selection, index test, and reference standard.

### 2.3 Statistical Analysis

The data were analyzed using Stata 18 (StataCorp LLC, College Station, TX, USA), Review Manager 5.4 (The Cochrane Collaboration, London, UK), and R 4.4.1 (R Foundation for Statistical Computing, Vienna, Austria) at the research level. We calculated the pooled relative risk (RR) for the involvement of various organs in systemic amyloidosis as assessed by PET, and separately calculated the pooled sensitivity, specificity, positive likelihood ratio (LR+), negative likelihood ratio (LR–), diagnostic odds ratio (DOR), and their respective 95% confidence intervals (CIs), as well as the area under the summary receiver operating characteristic (SROC) curve (AUC) for PET assessment of cardiac involvement. A continuity correction of 0.5 was applied for zero events in the studies. Heterogeneity was estimated using the I-squared (I^2^) index, which represents the percentage of variability between studies due to heterogeneity rather than chance. A random-effects model was used when the I^2^ statistic was >50%, while a fixed-effects model was used when the I^2^ statistic was <50%. A funnel plot was used to qualitatively assess potential publication bias, and Egger’s test was used to check for funnel plot asymmetry. Given the different radiopharmaceuticals and amyloidosis subtypes in the studies, a subgroup analysis was also performed on studies assessing cardiac involvement by PET. A two-sided *p*-value <0.05 was considered statistically significant.

## 3. Results

### 3.1 Study Selection and Characteristics

**Fig. 1.**
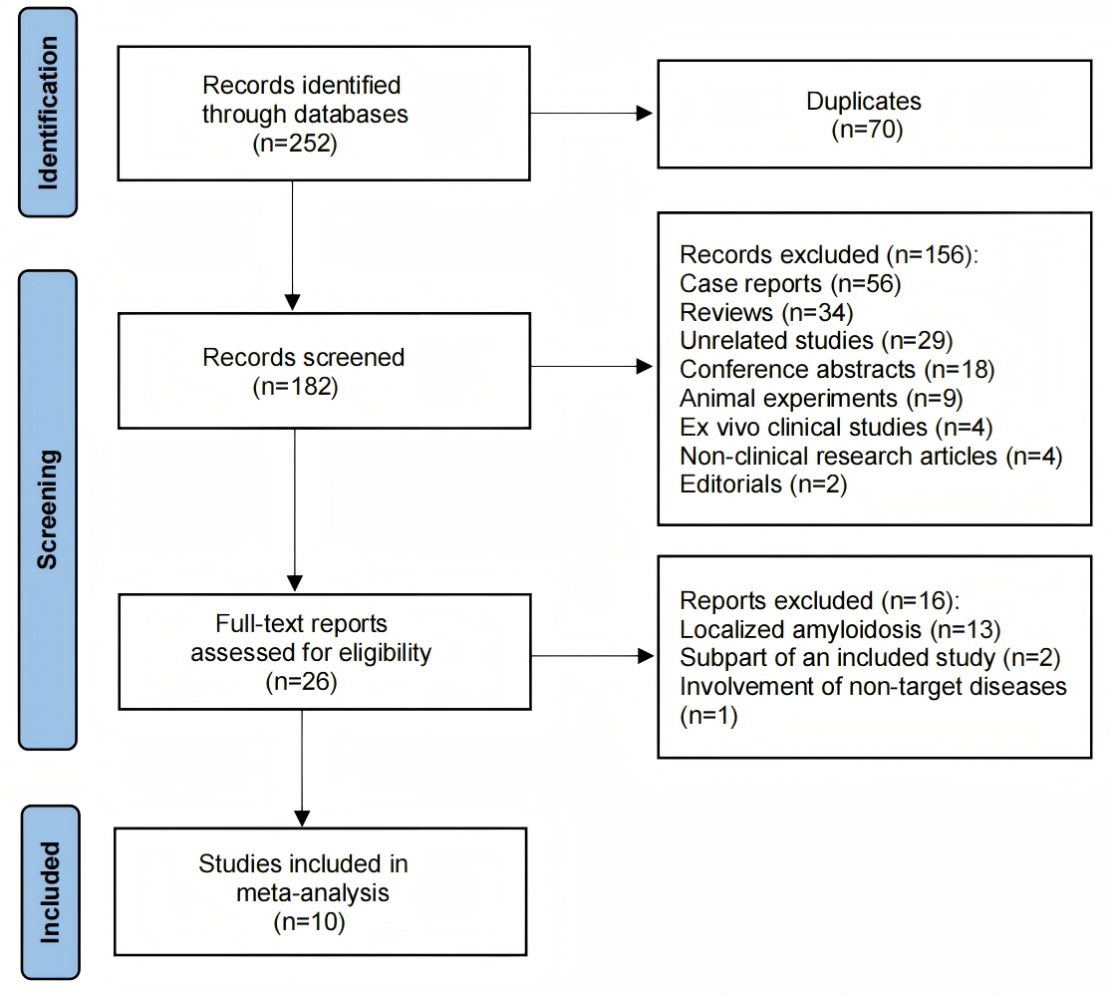
**Search results and flow chart of the meta-analysis**.

A total of 252 articles were identified from the searched databases. After removing duplicates, 182 articles remained. Following an initial screening, 156 studies were excluded. These included 29 unrelated studies, 56 case reports, 18 conference abstracts, 2 editorials, 34 reviews, 9 animal experiments, 4 *ex vivo* clinical studies, and 4 non-clinical research articles. After a full-text review of the remaining 26 articles, 16 were excluded for focusing on localized amyloidosis (n = 13), being a subpart of an already included study (n = 2), or involving non-target diseases (n = 1). Finally, 10 studies were selected and included in this study. The detailed process of study selection in the current meta-analysis is shown in Fig. 1. The characteristics of the included studies are shown in Table 1 (Ref. [6, 13, 14, 15, 16, 17, 18, 19, 20, 21]).

**Table 1.** **Characteristics of the included studies**.

Study	Year	No. of systemic amyloidosis	No. of AL	No. of ATTR	No. of controls	Modalities	Tracers
Antoni *et al*. [13]	2013	10	7	3	5	PET/CT	^11^C-PIB
Baratto *et al*. [20]	2018	7	7	NA	2	PET/MRI	^18^F-Florbetaben
Cuddy *et al*. [16]	2020	45	45	NA	NA	PET/CT	^18^F-Florbetapir
Ehman *et al*. [19]	2019	40	40	NA	NA	PET/CT	^18^F-Florbetapir
Ezawa *et al*. [6]	2018	15	7	8	3	PET/CT	^11^C-PIB
Manwani *et al*. [15]	2018	15	15	NA	NA	PET/CT	^18^F-Florbetapir
Mestre-Torres *et al*. [17]	2018	13	8	3	12	PET/CT	^18^F-Florbetapir
Wagner *et al*. [18]	2018	17	15	2	NA	PET/CT	^18^F-Florbetapir
Wall *et al*. [21]	2023	50	25	20	7	PET/CT	^124^I-Evuzamitide
Wang *et al*. [14]	2022	20	20	NA	3	PET/MRI	^11^C-PIB

AL, light chain amyloidosis; ATTR, transthyretin amyloidosis; PET/CT, positron emission tomography/computed tomography; ^11^C-PIB, ^11^C-Pittsburgh Compound B; NA, not applicable; PET/MRI, positron emission tomography/magnetic resonance imaging.

### 3.2 Quality Assessment

**Fig. 2.**
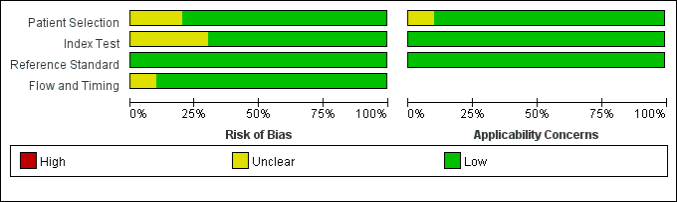
**Risk of bias and applicability concerns on the Quality Assessment of Diagnostic Accuracy Studies (QUADAS-2) tool of the enrolled studies**.

The summary of the risk of bias and applicability concerns, based on the modified QUADAS-2, is shown in Fig. 2. Overall, the quality of the included studies is considered satisfactory.

### 3.3 Diagnostic Performance of PET Imaging for Detection of Systemic Amyloidosis

**Fig. 3.**
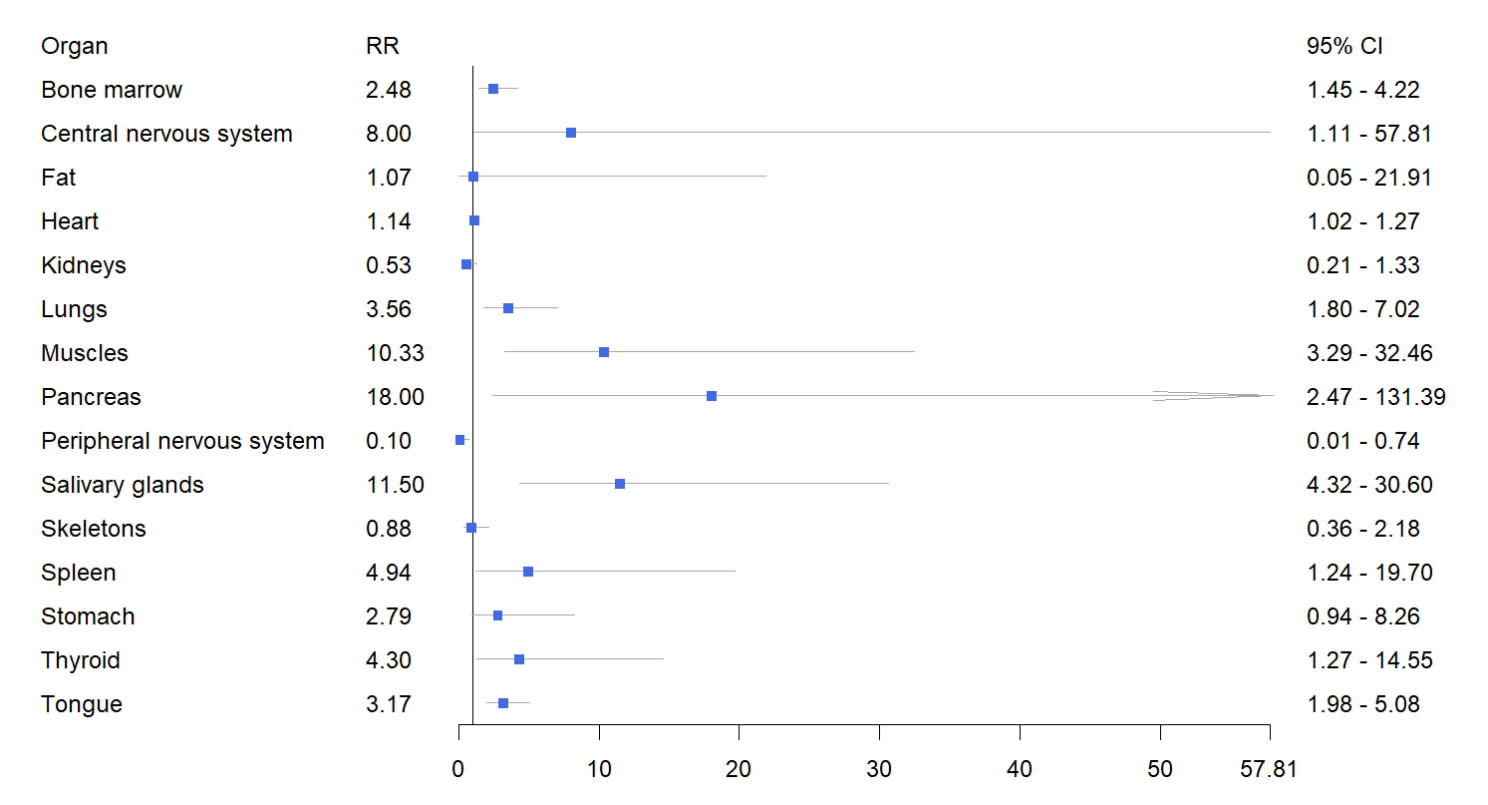
**Forest plot of the pooled relative risk of organ involvement in systemic amyloidosis detected by PET imaging**. RR, relative risk.

The relative risk and 95% confidence interval for organ involvement in systemic amyloidosis diagnosed by PET are as follows: bone marrow (2.48 (1.45, 4.22), *p* = 0.009), central nervous system (CNS) (8.00 (1.11, 57.81), *p* = 0.0393), heart (1.14 (1.02, 1.27), *p* = 0.0244), lungs (3.56 (1.80, 7.02), *p* = 0.0003), muscles (10.33 (3.29, 32.46), *p* < 0.0001), pancreas (18.00 (2.47, 131.39), *p* = 0.0044), salivary glands (11.50 (4.32, 30.60), *p* < 0.0001), spleen (4.94 (1.24, 19.70), *p* = 0.0236), thyroid (4.30 (1.27, 14.55), *p* = 0.0188), tongue (3.17 (1.98, 5.08), *p* < 0.0001). The overall detection rate of organ involvement in systemic amyloidosis assessed by PET was higher than that of clinical standards for the organs mentioned above, with a statistically significant difference (*p* < 0.05). The detection rates for the involvement of other organs are shown in Fig. 3 and Table 2.

**Table 2.** **Comparison of organ involvement detection by PET and clinical evaluation in systemic amyloidosis: pooled relative risk of PET vs. clinical standards**.

Organ	Clinical	PET	RR (95% CI)	*p*
Bone marrow	0.25 (0.09, 0.55)	0.64 (0.49, 0.76)	2.48 (1.45, 4.22)	0.009
Central nervous system	0.00 (0.00, 1.00)	0.37 (0.08, 0.81)	8.00 (1.11, 57.81)	0.0393
Fat	0.13 (0.07, 0.22)	0.10 (0.00, 0.73)	1.07 (0.05, 21.91)	0.964
Heart	0.65 (0.54, 0.74)	0.70 (0.47, 0.86)	1.14 (1.02, 1.27)	0.0244
Kidneys	0.45 (0.26, 0.65)	0.23 (0.11, 0.42)	0.53 (0.21, 1.33)	0.1766
Lungs	0.08 (0.04, 0.16)	0.33 (0.24, 0.43)	3.56 (1.80, 7.02)	0.0003
Muscles	0.01 (0.00, 0.07)	0.33 (0.17, 0.55)	10.33 (3.29, 32.46)	<0.0001
Pancreas	0.00 (0.00, 1.00)	0.20 (0.04, 0.58)	18.00 (2.47, 131.39)	0.0044
Peripheral nervous system	0.22 (0.02, 0.76)	0.00 (0.00, 1.00)	0.10 (0.01, 0.73)	0.0237
Salivary glands	0.03 (0.01, 0.09)	0.46 (0.30, 0.64)	11.50 (4.32, 30.60)	<0.0001
Skeletons	0.14 (0.07, 0.26)	0.12 (0.06, 0.24)	0.88 (0.36, 2.18)	0.7858
Spleen	0.01 (0.00, 0.28)	0.29 (0.22, 0.37)	4.94 (1.24, 19.70)	0.0236
Stomach	0.09 (0.01, 0.51)	0.45 (0.13, 0.82)	2.79 (0.94, 8.26)	0.0637
Thyroid	0.08 (0.04, 0.15)	0.35 (0.27, 0.44)	4.30 (1.27, 14.55)	0.0188
Tongue	0.16 (0.10, 0.24)	0.50 (0.41, 0.60)	3.17 (1.98, 5.08)	<0.0001

### 3.4 Diagnostic Performance of PET for Detecting Cardiac Involvement in Systemic Amyloidosis

**Fig. 4.**
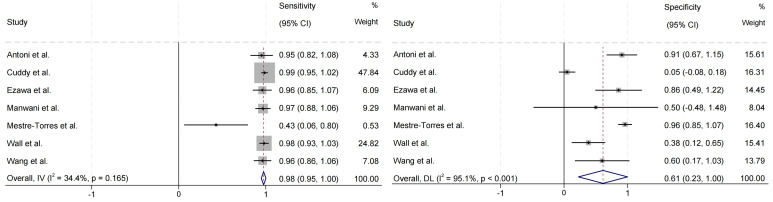
**Forest plot of pooled sensitivity and specificity of cardiac involvement in systemic amyloidosis detected by PET imaging**. IV, inverse variance; DL, DerSimonian and Laird.

**Fig. 5.**
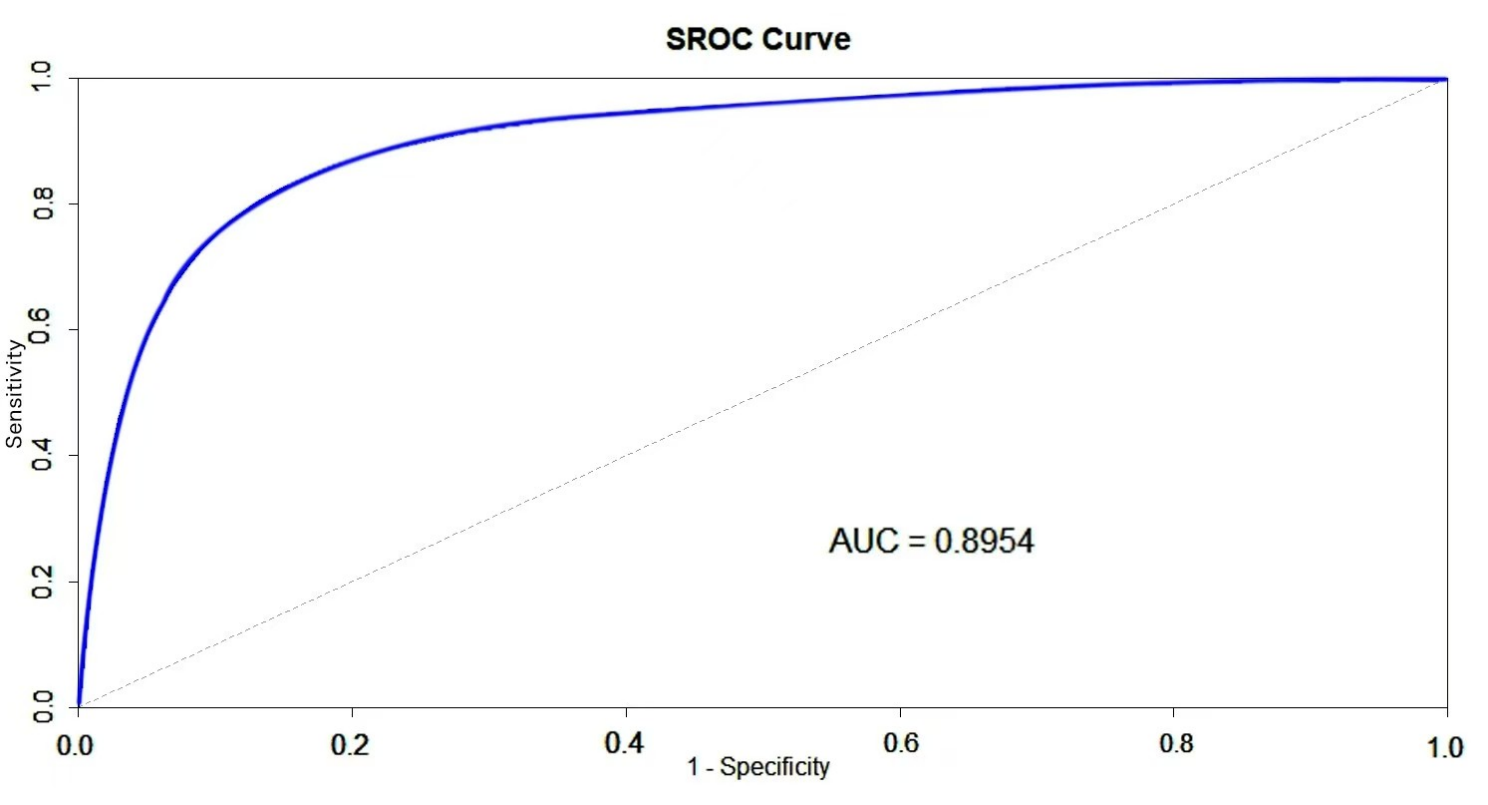
**Summary receiver operating characteristic (SROC) curve for diagnosis of cardiac involvement in systemic amyloidosis detected by PET imaging**. AUC, area under the curve.

The forest plot of the sensitivity and specificity of PET in diagnosing cardiac involvement in systemic amyloidosis is shown in Fig. 4. Among the seven studies that evaluated cardiac involvement, the pooled sensitivity was 0.98 (95% CI = (0.95, 1.00)), the pooled specificity was 0.61 (95% CI = (0.23, 1.00)), and the pooled LR+, LR–, and DOR were 4.70 (95% CI = (2.98, 6.42)), 0.07 (95% CI = (0.01, 0.14)), and 219.05 (95% CI = (–142.59, 580.69)), respectively. The SROC in Fig. 5 shows an AUC of 0.8954.

### 3.5 Subgroup Analysis of Different Radiotracers

The number of studies that conducted PET imaging analysis using the radiotracers ^11^C-PIB, ^18^F-Florbetapir, and ^124^I-Evuzamitide were 3, 3, and 1, respectively. The pooled sensitivities for the ^11^C-PIB and ^18^F-Florbetapir subgroups were 0.96 (95% CI = (0.90, 1.02)) and 0.93 (95% CI = (0.80, 1.05)), respectively. The pooled specificities for the ^11^C-PIB and ^18^F-Florbetapir subgroups were 0.84 (95% CI = (0.66, 1.02)) and 0.50 (95% CI = (0.27, 1.27)), respectively. The sensitivity and specificity for the ^124^I-Evuzamitide subgroup were 0.98 and 0.38, respectively. Table 3 presents a comparison of the diagnostic performance of PET imaging for cardiac involvement using different radiotracers.

**Table 3.** **Comparison of the diagnostic performance of PET imaging for cardiac involvement using different radiotracers**.

	All studies	^11^C-PIB	^1^^8^F-Florbetapir	^124^I-Evuzamitide
Sensitivity	0.98	0.96	0.93	0.98
I^2^	34.4%	0.0%	77.4%	NA
*p*-value	0.165	0.992	0.012	NA
Specificity	0.61	0.84	0.50	0.38
I^2^	95.1%	0.0%	98.2%	NA
*p*-value	<0.001	0.466	<0.001	NA
LR+	4.7	6.44	4.55	1.59
I^2^	97.7%	94.9%	98.9%	NA
*p*-value	<0.001	<0.001	<0.001	NA
LR–	0.07	0.06	0.29	0.05
I^2^	21.4%	0.0%	66.4%	NA
*p*-value	0.267	0.959	0.051	NA
DOR	219.05	7.84	476.68	31.25
I^2^	100.0%	95.1%	100.0%	NA
*p*-value	<0.001	<0.001	<0.001	NA

DOR, diagnostic odds ratio; LR+, positive likelihood ratio; LR–, negative likelihood ratio.

### 3.6 Subgroup Analysis of AL and ATTR

**Fig. 6.**
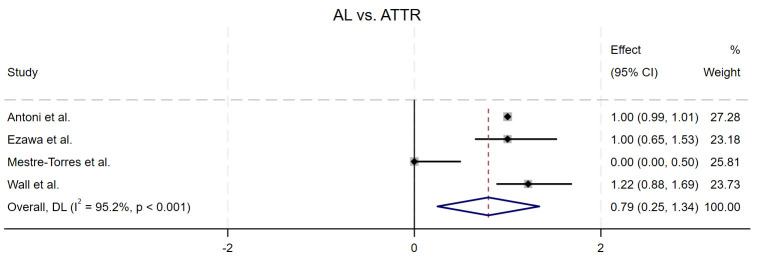
**Forest plot of the pooled relative risk of AL vs ATTR detecting cardiac involvement, AL showed a significantly higher RR than that for ATTR**.

The pooled RR for four studies comparing ATTR to AL was calculated as 0.79 (95% CI = (0.25, 1.34)), with a *p*-value of 0.004, suggesting statistical significance. However, the confidence interval crosses 1, which may weaken the significance of the result. The I^2^ statistic was 95.2%, indicating substantial heterogeneity between the study results (Fig. 6).

### 3.7 Publication Bias

**Fig. 7.**
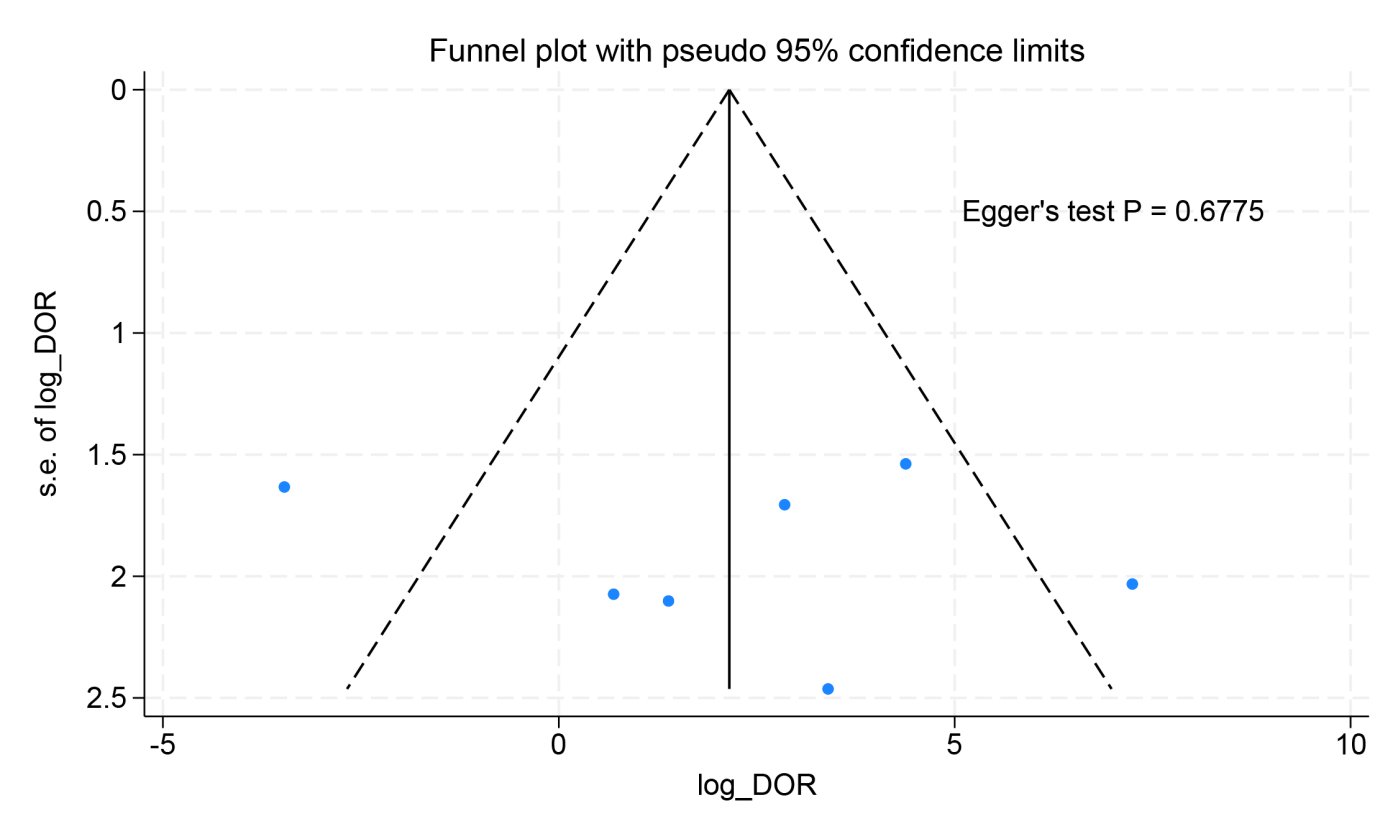
**Funnel plot results for the diagnostic performance of PET imaging detecting cardiac involvement**.

To assess potential publication bias, the funnel plot was designed, and Egger’s test was performed. Visual inspection of the funnel plot revealed no evidence of publication bias in studies assessing the diagnostic performance of PET for cardiac involvement. Egger’s test also showed no evidence of asymmetry in the funnel plots (Fig. 7).

## 4. Discussion

This study, through a meta-analysis of published literature, found that PET imaging has high diagnostic value in detecting amyloid deposition in various organs affected by systemic amyloidosis, particularly in the detection of cardiac involvement, showing high sensitivity and good predictive ability.

In the 10 studies included, PET demonstrated statistically significant results in detecting amyloid deposition in multiple organs, including the bone marrow, CNS, heart, lungs, muscles, pancreas, salivary glands, spleen, thyroid, and tongue. Compared to traditional clinical diagnostic methods, PET showed higher detection rates for organ involvement. This provides strong support for the widespread use of PET in the evaluation of systemic amyloidosis.

PET imaging is particularly advantageous in assessing cardiac, pulmonary, splenic, bone marrow, glandular, and soft tissue involvement, while it has certain limitations in evaluating the peripheral nervous system and kidneys. The kidney, as one of the most commonly affected organs with 50% to 80% of systemic amyloidosis patients exhibiting renal involvement [22], often presents as nephrotic syndrome [23, 24], and in severe cases, can progress to end-stage kidney disease (ESKD), requiring renal replacement therapies such as dialysis or kidney transplantation [25]. However, PET imaging is less effective in detecting kidney involvement. This limitation may be due to the high metabolic activity of the kidneys as excretory organs, which could obscure the pathological changes caused by amyloidosis, thereby affecting PET’s sensitivity in detecting renal involvement.

Cardiac involvement is one of the most critical prognostic factors in patients with systemic amyloidosis [26], with approximately 61% of these patients dying from CA [27]. The seven studies included in this meta-analysis regarding cardiac involvement indicated that PET has high sensitivity (0.98) and moderate specificity (0.61) in diagnosing CA, with an AUC of 0.8954. These findings suggest that PET has significant potential in screening for CA. Specifically, ^124^I-Evuzamitide exhibited high sensitivity (0.98), while ^11^C-PIB showed high specificity (0.84). These results indicate that different radiotracers have varying performances in detecting CA, and clinicians can choose the best tracer based on specific circumstances. ^11^C-PIB, a derivative of the amyloid-binding dye thioflavin-T, binds to β-amyloid plaques, allowing clear visualization of amyloid deposition in the brain on PET scans. It is believed to bind with various types of amyloid fibrils, making it highly promising for imaging organ involvement in systemic amyloidosis [28]. However, the short half-life of ^11^C (20 minutes) limits its practical use in routine clinical applications [29]. In contrast, ^18^F-labeled amyloid PET radiotracers, such as ^18^F-Florbetapir, have longer half-lives and offer significant clinical advantages. A study by Clerc *et al*. [30] evaluated the prognostic value of ^18^F-Florbetapir PET imaging quantifying left ventricular amyloid load in systemic light chain amyloidosis. They found that left ventricular amyloid burden was closely associated with the occurrence of major adverse cardiac events, further emphasizing the potential of ^18^F-Florbetapir in assessing patient prognosis. Additionally, ^124^I-Evuzamitide, a novel broad-spectrum amyloid radiotracer, has also shown substantial potential in imaging CA. Research has shown that ^124^I-Evuzamitide can accurately differentiate CA from controls, with performance similar to ^18^F-Florbetapir in AL-CA and possibly more advantageous in wild-type transthyretin CA. Moreover, ^124^I-Evuzamitide correlates strongly with cardiac structural and functional indices, further confirming its potential in detecting and quantifying cardiac amyloid deposits [31]. CA often presents as restrictive cardiomyopathy, and its early symptoms are subtle, often going unnoticed. As a non-invasive imaging technique, PET can detect cardiac involvement through whole-body scans, which is crucial for early diagnosis and the formulation of personalized treatment plans.

In subgroup analysis, we compared the effectiveness of PET in detecting cardiac involvement in AL and ATTR amyloidosis patients. The results showed that the RR in AL patients was significantly higher than in ATTR patients (*p* = 0.004), indicating that PET is more sensitive in diagnosing cardiac involvement in AL amyloidosis. This difference may be related to the heterogeneity of amyloid deposition in AL and ATTR types and their differing invasiveness in the heart. Despite the statistical significance of the subgroup analysis, there was considerable heterogeneity in the data (I^2^ = 95.2%), suggesting that results varied widely across studies. Therefore, further research is needed to explore the differences in PET diagnostic performance between different subtypes of amyloidosis.

Previous studies have conducted meta-analyses to assess the diagnostic accuracy of detecting CA. A meta-analysis of six studies involving bone scintigraphy in ATTR-CA patients (529 patients) showed high sensitivity (92.2%, 95% CI = (89%, 95%)) and specificity (95.4%, 95% CI = (77%, 99%)), with an LR+ of 7.02 (95% CI = (3.42, 14.4)), an LR– of 0.09 (95% CI = (0.06, 0.14)), and a DOR of 81.6 (95% CI = (44, 153)) [32]. Kim *et al*. [33] conducted a meta-analysis of six PET imaging studies for CA (98 patients) and found a pooled sensitivity of 0.95, specificity of 0.98, LR+ of 10.130, LR– of 0.1, and DOR of 148.83. Additionally, semi-quantitative parameters of amyloid proteins in PET imaging demonstrated a diagnostic advantage for AL amyloidosis over ATTR amyloidosis. Another meta-analysis of 13 studies (90 patients) showed a pooled sensitivity of 0.97 and specificity of 0.98 for amyloid PET. The pooled sensitivity of F-18 labeled NaF PET was 0.63, with specificity of 1.00. Combining amyloid PET with F-18 labeled NaF PET showed a sensitivity of 0.88 and specificity of 0.98 [34]. A meta-analysis of non-invasive myocardial imaging for CA, including cardiac magnetic resonance (CMR), single photon emission computed tomography (SPECT), and PET, reported sensitivities of 0.84, 0.98, and 0.78, respectively, with specificities of 0.87, 0.92, and 0.95. SPECT demonstrated better diagnostic performance than the other two techniques in detecting CA [35].

Although this meta-analysis comprehensively assessed the diagnostic performance of PET in systemic amyloidosis, several limitations remain. First, the number of studies included was relatively small (only 10 studies), and many had small sample sizes, which may affect the robustness and external validity of the results. Second, the use of different radiotracers and the heterogeneity of amyloidosis subtypes increased the complexity of interpreting the results. High statistical heterogeneity in some pooled estimates introduces uncertainty and warrants cautious interpretation of these results. Therefore, future high-quality, large-scale prospective studies are needed to further validate the diagnostic efficacy of PET in different subtypes of systemic amyloidosis. While PET can detect amyloid deposits in multiple organs, how to integrate it with other imaging modalities (such as echocardiography, MRI, etc.) and biomarkers to form a more accurate diagnostic strategy remains an unresolved issue in clinical practice.

## 5. Conclusion

In conclusion, PET imaging has significant clinical value in diagnosing systemic amyloidosis, particularly in the early detection of cardiac involvement. By using different radiotracers, PET provides a more comprehensive and accurate whole-body evaluation for patients with amyloidosis, helping clinicians make more informed diagnostic and treatment decisions in the early stages of the disease. With the continuous emergence of novel radiotracers and the advancement of high-quality studies, the clinical applications of PET in systemic amyloidosis are expected to become even more promising.

## Data Availability

The datasets used and analyzed during the current study are available from the corresponding author on reasonable request.

## Abbreviations

ATTR, transthyretin amyloidosis; AL, light chain amyloidosis; CA, cardiac amyloidosis; MRI, magnetic resonance imaging; NT-proBNP, N-terminal pro-B-type natriuretic peptide; PYP, pyrophosphate; DPD, 3,3-diphospho-1,2-propanodicarboxylic acid; HMDP, hydroxymethylene diphosphonate; SAP, serum amyloid P component; PET, positron emission tomography; ^11^C-PIB, ^11^C-Pittsburgh Compound-B; QUADAS-2, Quality Assessment of Diagnostic Accuracy Studies; RR, relative risk; LR+, positive likelihood ratio; LR–, negative likelihood ratio; DOR, diagnostic odds ratio; CIs, confidence intervals; SROC, summary receiver operating characteristic; AUC, area under the curve; I^2^, I-squared; CNS, central nervous system; ESKD, end-stage kidney disease; NaF, sodium fluoride; SPECT, single photon emission computed tomography.

## Supplementary Material

Supplementary material associated with this article can be found, in the online version, at https://doi.org/10.31083/RCM37731.
